# Detoxification of LTA by intracanal medication: analysis by macrophages proinflammatory cytokines production

**DOI:** 10.1590/0103-6440202205195

**Published:** 2022-12-05

**Authors:** Luciane Dias de Oliveira, Felipe Eduardo de Oliveira, Bárbara Araujo Hatje, Marcia Carneiro Valera, Cláudio Antonio Talge Carvalho, Amjad Abu Hasna

**Affiliations:** 1 Department of Bioscience and Oral Diagnosis, Institute of Science and Technology, São Paulo State University - UNESP, São José dos Campos, São Paulo, Brazil; 2 Department of Restorative Dentistry, Endodontics division, Institute of Science and Technology, São Paulo State University - UNESP, São José dos Campos, São Paulo, Brazil.

**Keywords:** lipoteichoic acid, cytokines, macrophages, calcium hydroxide, chlorhexidine

## Abstract

The aim of this study was to evaluate *in vitro* the effect of calcium hydroxide [Ca(OH)_2_], 2% chlorhexidine gel (CHX) on macrophages (RAW 264.7) to produce pro-inflammatory cytokines and nitric oxide after pretreatment with lipoteichoic acid (LTA) of *Enterococcus faecalis*. Forty-eight human single-rooted teeth were instrumented with R25.08 (RECIPROC) and sterilized by gamma irradiation. LTA was inoculated in the root canal of each specimen for 96 hours. Specimens were instrumented with 40.06 and 50.05 (RECIPROC) and medicated with: I) Pyrogen-free saline solution (SS); II) 2% CHX gel; III) Ca(OH)_2_ + SS; or IV) Ca(OH)_2_ + CHX for 14 days. Three samples (S) were performed of the root canal of each specimen at: S1) immediately after instrumentation; S2) after Ethylenediaminetetraacetic acid (EDTA); S3) after intracanal medication removal. Subsequent quantification of cytokines (IL-1β, TNF-α, MIP-1α, IP-10, G-CSF and IL-6) by immunosorbent assay (ELISA) and nitric oxide by the Griess method was carried-out. Data were submitted to a normality test and then analyzed with one-way ANOVA and Tukey test with a significance level of 5% using GraphPad Prism 6. Ca(OH)_2_ + SS and Ca(OH)_2_ + CHX presented lower levels of TNF-α, TNF-α, IL-6, G-CSF and nitric oxide. Ca(OH)_2_ + SS was the most effective in reducing MIP-1α. CHX was effective in reducing IL-6 and G-CSF. Therefore, the combined intracanal medication of calcium hydroxide and chlorhexidine is effective in reducing the cytokines TNF-α, IL-1β, IL-6, G-CSF and nitric oxide.

## Resumo

O objetivo deste estudo foi avaliar in vitro o efeito do hidróxido de cálcio [Ca(OH)_2_], clorexidina gel a 2% (CHX) em macrófagos (RAW 264.7) na produção de citocinas pró-inflamatórias e óxido nítrico após pré-tratamento com ácido lipoteicóico (LTA) de *Enterococcus faecalis*. Quarenta e oito dentes humanos uniradiculares foram instrumentados com R25.08 (RECIPROC) e esterilizados por irradiação gama. LTA foi inoculado no canal radicular de cada espécime por 96 horas. Os espécimes foram instrumentados com 40.06 e 50.05 (RECIPROC) e medicados com: I) solução salina apirogênica (SS); II) CHX 2%; III) Ca(OH)_2_ + SS; ou IV) Ca(OH)_2_ + CHX durante 14 dias. Três amostras (S) foram realizadas do canal radicular de cada espécime em: S1) imediatamente após a instrumentação; S2) após ácido etilenodiaminotetracético (EDTA); S3) após a retirada da medicação intracanal. Foi realizada a quantificação subsequente de citocinas (IL-1β, TNF-α, MIP-1α, IP-10, G-CSF e IL-6) por imunoensaio (ELISA) e de óxido nítrico pelo método de Griess. Os dados foram submetidos a um teste de normalidade e então analisados com ANOVA one-way e teste de Tukey com nível de significância de 5% usando GraphPad Prism 6. Ca(OH)_2_ + SS e Ca(OH)_2_ + CHX apresentaram níveis mais baixos de TNF -α, TNF-α, IL-6, G-CSF e óxido nítrico. Ca(OH)_2_ + SS foi o mais eficaz na redução de MIP-1α. CHX foi eficaz na redução de IL-6 e G-CSF. Sendo assim a associação de hidróxido de cálcio e clorexidina é eficaz na redução das citocinas TNF-α, IL-1β, IL-6, G-CSF e óxido nítrico.

## Introduction

Endodontic infection has a complex nature as bacteria, endotoxins, matrix metalloproteinases and cytokines have etiological role in its development ^1^. Although, it is well known that Gram-negative bacteria like *Porphyromonas*, *Prevotella*, *Fusobacterium*, *Campylobacter*, *Tannerella*, *Treponema* and *Dialister* are predominant in primary endodontic infections, and Gram-positive bacteria like *Enterococcus*, *Actinomyces*, *Streptococcus*, *Lactobacillus*, *Candida*, *Propionibacterium*, *Staphylococcus*, *Eubacterium* and *Bifidobacterium* are predominant in secondary endodontic infections, still, both Gram-positive and Gram-negative bacteria are found in both types of endodontic infections ^2^. 

*Enterococcus faecalis*, a Gram-positive bacterium, resists several antimicrobial agents because of its virulence factors including lipoteichoic acid (LTA), gelatinase, hyaluronidase, cytolysin, aggregation substance, pheromones and heat shock proteins ^3^. LTA is an endotoxin pf Gram-positive bacteria like *E. faecalis*, it is a chemical substance found in the outer membrane of bacterial cell ^4^, differently, the endotoxin of Gram-negative bacteria is known as lipopolysaccharide (LPS). Endotoxins can be released during the duplication or death of these bacteria in infected root canals and has a role in developing periapical lesions ^5^. In the literature, LPS effect over macrophages, the predominant defense cell in periapical lesions have been studied to understand the production of inflammatory mediators ^6^. Conversely, LTA effect is little explored ^7^.

LTA is similar to LPS in structure and immunological properties ^8^. It has amphipathic structure formed by linking the poly glycerophosphates to fatty acids and it is involved in inflammatory response, septic syndrome, biofilm formation and adhesion to dentin surface ^8^. It can activate macrophages and induce the release of nitric oxide and proinflammatory cytokines such as IL-1β, IL-6 and TNF-α ^9^ ^,^ ^10^. It promotes cell activation via TLR-2 receptor differently of LPS that promotes by signaling via TLR-4 receptor ^9^. It can accumulate where *E. faecalis* resides as in dentin and periapical lesions ^8^ and has a role in pulp tissue destruction, therefore, it is necessary to understand its role in inflammations of dental pulp and periapical lesions ^7^ ^,^ ^11^. 

Calcium hydroxide [Ca (OH)_2_] is a widely used intracanal medication because of its antimicrobial action and biocompatibility besides other properties ^12^. Alternatively, chlorhexidine (CHX) is another intracanal medication, it is effective over variety of microorganisms and is biocompatible ^13^. Both Ca (OH)_2_ and CHX are effective over endotoxins ^11^. However, their combined effect is still questionable ^14^.

The aim of this study was to evaluate *in vitro* the effect of different intracanal medications (calcium hydroxide, 2% chlorhexidine gel and calcium hydroxide combined with 2% chlorhexidine gel) on macrophages (RAW 264.7) to produce pro-inflammatory cytokines (IL-1β, TNF-α, MIP-1α, IP-10, G-CSF and IL-6) and nitric oxide after pretreatment with LTA of *E. faecalis*. The null hypothesis is that calcium hydroxide, chlorhexidine gel, and its combination have no effect on the production of pro-inflammatory cytokines and nitric oxide by macrophages.

## Material and methods

The research ethics committee involving human beings of São Paulo State University (n 149.301) authorized this study. An informed consent form was obtained of each patient declaring conscience about the use of his/her extracted teeth in this in vitro study.

### Preparation of specimens

Forty-eight freshly extracted human single-rooted teeth (caries- and calcification-free) were used. The teeth were selected basing on dimensional and morphological similarities. The crown of each tooth was cross-cut to standardize a length of 16±0.5 mm of all specimens. R25.08 (RECIPROC, VDW, Germany) was used for instrumentation at working length of 15 mm to standardize the root canal diameter of all specimens. Saline solution (SS) was used as irrigant (5 mL for each third of the root canal). Then, the canals were filled with 17% ethylenediaminetetraacetic acid (EDTA) (Porto Alegre, RS, Brazil) for 3 min and washed with 10 mL of SS and dried-out with sterile paper points 25.08 (RECIPROC, VDW, Germany). The apical region of each specimen was sealed using light cured composite resin (Admira Fusion, Voco, Cuxhaven, Germany), then, the root external surface was coated with two layers of adhesive epoxy, except the region of the cervical opening ^12^. Lastly, the specimens were randomly distributed and fixed with chemically activated acrylic resin (JET, Artigos Odontológicos Clássico, Campo Limpo Paulista, SP, Brazil) in four different 24-wells microplates (TPP, Zollstrasse, Switzerland) considering (n=12). All microplates and materials used in this study (files, tweezers, spatulas, scissors, gloves, etc.) were sterilized by gamma irradiation with cobalt 60 (20 kGy for 6 hours) to neutralize preexisting endotoxins.

LTA of *E. faecalis* (Sigma-Aldrich, St. Louis, MO, USA) was standardized in double distilled pyrogen-free water at a concentration of 250 µg/mL. The standardized LTA solution (three aliquots of 10 µL) was inoculated in the root canal of each specimen every 24 h totaling three cycles of inoculation in 72 hours. All specimens were kept at 37°C with relative humidity. After 24 hours of the last inoculation, all specimens were instrumented with 40.06 and 50.05 (RECIPROC, VDW, Germany). Pyrogen-free SS (Aster) (5 mL for each third of the root canal) was used as irrigant. Then, the canals were filled with EDTA 17% for 3 min and washed with 10 mL of pyrogen-free SS and dried-out with sterile paper points 50.05 (RECIPROC, VDW, Germany).

### Experimental groups

Randomly, the microplates were divided into four experimental groups (n=12) and medicated using one of the following intracanal medication:

Group 1 (SS): Pyrogen-free saline solution was used to fill the entire root canal of the specimens (Control group).

Group 2 (CHX): 2% CHX gel (Biofórmula Manipulação, São José dos Campos, SP, Brazil) was used to fill the entire root canal of the specimens.

Group 3 (Ca (OH)_2_+ SS): The powder of Ca (OH)_2_(Biodynamics Chemicals and Pharmaceuticals LTDA, Paraná, Brazil) was manipulated with pyrogen-free SS over a sterilized glass plate (by radiation) until obtaining a smooth paste with no lumps. The paste consistency was toothpaste-like where then it was used to fill the entire root canal of the specimens. The proportion of the paste was 1 g of Ca (OH)_2_powder with 1 mL of pyrogen-free SS

Group 4 (Ca (OH)_2_ + CHX): The powder of Ca (OH)_2_was manipulated with 2% CHX gel over a sterilized glass plate until obtaining a smooth paste with no lumps. The paste consistency was toothpaste-like where then it was used to fill the entire root canal of the specimens. The proportion of the paste was 1 g of Ca (OH)_2_powder with 1 mL of 2% CHX gel.

All intracanal medications were inserted with Lentulo spiral. The specimens were kept at 37°C and relative humidity of 100% for 14 days. Then, the medications were removed using 10 mL of pyrogen-free SS.

Three samples (S) were performed of the root canal of each specimen at: S1) immediately after instrumentation; S2) after EDTA; S3) after intracanal medication removal. To collect a sample, each root canal was flooded with pyrogen-free SS, and a sample of 100 µL was collected of the root canal of each specimen using an insulin syringe and needle.

### Quantification of cytokines and nitric oxide

Cultures of Murine macrophages (RAW 264.7) (Rio de Janeiro Cell Bank-APABCAM-RJ, Brazil) were used in this study. The cells were grown in Dulbecco’s Modified Eagle Medium (DMEM) (LGC Biotechnology, Cotia, Brazil) supplemented with 10% fetal bovine serum (FBS) (Invitrogen, New York, USA), incubated at 37°C, with atmospheric humidity, with 5% CO_2_using cell culture flasks (TPP, Zollstrasse, Switzerland). To quantify the number of viable cells, the Trypan blue (0.4%, Sigma-Aldrich, St. Louis, MO, USA) exclusion test was performed. The cells were cultivated in 24-well microplates, 200 μl of DMEM medium was added and supplemented with 10% FBS containing 5 × 10^5^ viable cells. These plates were incubated (37°C, 5% CO_2_) for 24 hours for cell adhesion. Then, these cells were activated with 30 µl of each sample collected from root canals and incubated (37°C, 5% CO_2_) for 24 hours. Later, supernatants were removed and frozen (-20ºC) for subsequent detection and quantification of cytokines (IL-1β, TNF-α, MIP-1α, IP-10, G-CSF and IL-6) by immunosorbent assay (ELISA) and nitric oxide by the Griess method.

Anti-IL-1β, anti-TNF-α, anti-MIP-1α anti-IP-10, anti-G-CSF and anti-IL-6 (R & D Systems, NS) DuoSet kits were used to perform ELISA assay according to the manufacturer recommendations. After obtaining the optical densities, the levels of cytokines (pg/mL) present in the culture supernatants of macrophages were determined using GraphPad Prism 5.0.

Nitric oxide production in the culture supernatants of macrophages was determined indirectly by the nitrite concentration detected by the Griess reagent (Sigma-Aldrich, St. Louis, MO, USA) in comparison with the standard curve nitrite (Sigma-Aldrich, St. Louis, MO, USA). The plates were read in a microplate reader (BIO-TEK Instruments, Highland Park, Winooski, VT, USA), and the optical densities were calculated at a wavelength of 520 nm.

### Statistical analysis

Data were submitted to a normality test and then analyzed with one-way ANOVA and Tukey test with a significance level of 5% using GraphPad Prism 6 (La Jolla, CA, USA).

## Results

### TNF-α

It was found that the production of TNF-α levels was increased significantly after intracanal medication (S3) in all groups when compared to S1 and S2 (p<0.05). However, intergroup comparison showed a statistically significant difference (p<0.05) between Ca (OH)_2_ + SS and Ca (OH)2 + CHX on one side (lower levels of TNF-α production and with the control and CHX groups on the other side (higher levels of TNF-α production) in S3. Still, CHX had a statistically significant difference when compared to the control group in S3 (Table 1). 

### IL-1β

It was found that the production of IL-1β levels was increased significantly after intracanal medication (S3) in all groups when compared to S1 and S2 (p<0.05) except of Ca (OH)_2_ + SS group (p>0.05). Besides, there was a significant difference (p<0.05) among the groups in S3, in which Ca (OH)_2_ + SS and Ca (OH)_2_ + CHX presented lower levels of IL-1β in comparison with the control and CHX groups. Still, the control group presented a lower level of IL-1β production being different (p<0.05) of CHX group (Table 1).

**Table 1:** Mean values, standard deviation and formation of homogeneous groups of the production of cytokines TNF-α and IL-1β (pg/mL).

Groups	Mean values ± SD (pg/mL)					
	TNF-α			IL-1β		
	S1	S2	S3	S1	S2	S3
Control group	2.13 ± 2.67 a	0.00 ± 0.00 a	722.21 ± 379.95 A - b	2.18 ± 2.27 a	0.63 ± 1.29 a	4.65 ± 2.43 A - b
CHX	2.64 ± 3.20 a	2.37 ± 2.20 a	515.36 ± 427.13 B - b	1.56 ± 1.96 a	2.50 ± 2.58 a	10.02 ± 3.52 B - b
Ca (OH)_2_ + SS	3.67 ± 3.32 a	1.44 ± 2.39 a	198.68 ± 152.55 C - b	0.40 ± 1.27 a	1.72 ± 2.18 a	0.64 ± 1.69 C - a
Ca (OH)_2_ + CHX	3.25 ± 7.25 a	0.00 ± 0.00 a	59.36 ± 22.56 C - b	0.00 ± 0.00 a	0.00 ± 0.00 a	2.68 ± 2.51 C - b

Ca (OH)_2_: calcium hydroxide; SS: saline solution CHX: 2% chlorhexidine gel; Control group: pyrogen-free saline solution; S: Sample; SD: standard deviation. Different uppercase letters indicate inter-groups statistically significate differences (comparison in the same column), while different lowercase letters indicate intra-groups statistically significate differences (comparison in the same line).

### IL-6

It was found that the production of IL-6 levels was increased significantly after intracanal medication (S3) in all groups when compared to S1 and S2 (p<0.05) except of Ca (OH)_2_ + SS group (p>0.05). There was no significant difference (p>0.05) among Ca (OH)2 + SS, Ca (OH)2 + CHX and CHX groups, however, all were different in comparison with the control group (p<0.05) as they presented lower levels of IL-6 production (Table 2).

### Granulocyte colony stimulating factor (G-CSF)

There was no a significant difference (p>0.05) among S1, S2 and S3 in relation to G-CSF levels, regardless the intracanal medication used, except of the control group (p<0.05). Furthermore, there was no significant difference (p>0.05) among Ca (OH)2 + SS, Ca (OH)2 + CHX and CHX groups, however, all were different in comparison with the control group (p<0.05) as they presented lower levels of G-CSF production (Table 2).

**Table 2:** Mean values, standard deviation and formation of homogeneous groups of the production of cytokines IL-6 G-CSF (pg/mL).

Groups	Mean values ± SD (pg/mL)					
	IL-6			G-CSF		
	S2	S3	S1	S2	S3	S3
Control group	1.33 ± 1.36 a	0.14 ± 0.45 a	76.91 ± 83.76 A - b	25.58 ± 14.87 a	14.21 ± 12.77 a	1768.69 ± 640.36 A - b
CHX	1.85 ± 1.40 a	2.12 ± 0.72 a	17.11 ± 11.68 B - b	8.45 ± 5.81 a	12.30 ± 12.28 a	48.29 ± 88.38 B - a
Ca (OH)_2_ + SS	0.81 ± 1.11 a	0.48 ± 0.55 a	1.93 ± 2.06 B - a	2.01 ± 4.75 a	7.39 ± 6.54 a	8.48 ± 12.61 B - a
Ca (OH)_2_ + CHX	0.17 ± 0.49 a	0.01 ± 0.06 a	3.91 ± 1.81 B - b	8.10 ± 1.06 a	9.76 ± 2.96 a	10.89 ± 3.20 B - a

Ca (OH)_2_: calcium hydroxide; SS: saline solution CHX: 2% chlorhexidine gel; Control group: pyrogen-free saline solution; S: Sample; SD: standard deviation. Different uppercase letters indicate inter-groups statistically significate differences (comparison in the same column), while different lowercase letters indicate intra-groups statistically significate differences (comparison in the same line).

### MIP-1α (macrophage inflammatory protein-1α)

It was found that the production of MIP-1α levels was increased significantly after intracanal medication (S3) in all groups when compared to S1 and S2 (p<0.05) except of Ca (OH)_2_ + SS group (p>0.05) that induced a lower production of MIP-1α in comparison with S1 and S2. Besides, Ca (OH)_2_ + SS group as S3 induced a lower production of MIP-1α being significantly different (p <0.05) in comparison with the other groups (Table 3).

### Nitric oxide

It was found that the production of nitric oxide levels was increased significantly (p<0.05) after intracanal medication (S3) in the control and CHX groups when compared to S1 and S2, conversely, Ca (OH)_2_ + SS and Ca (OH)_2_ + CHX groups induced a lower production without a significant difference (p>0.05) of nitric oxide levels in comparison with S1 and S2 (Table 3). Intragroup comparison showed that Ca (OH)_2_ +SS and Ca (OH)_2_ + CHX presented lower levels of nitric oxide in comparison with the control and CHX groups. Still, the control group presented a higher level of nitric oxide production being different (p<0.05) of CHX group (Table 3).

**Table 3:** Mean values, standard deviation and formation of homogeneous groups of the production of cytokine MIP-1α and nitric oxide (pg/mL).

Groups	Mean values ± SD (pg/mL)					
	MIP-1α			Nitric oxide		
	S2	S3	S1	S2	S3	S3
Control group	10.64 ± 1.8 a	29.46 ± 20.3 a	91.92 ± 25.1 A - b	0.36 ± 0.12 a	0.22 ± 0.10 a	0.54 ± 0.19 A - b
CHX	14.36 ± 4.7 a	13.03 ± 9.3 a	110.38 ± 11.3 A - b	0.24 ± 0.12 a	0.22 ± 0.05 a	0.37 ± 0.12 B - b
Ca (OH)_2_ + SS	21.43 ± 7.7 a	25.90 ± 6.7 a	6.14 ± 2.37 B - a	0.25 ± 0.14 a	0.13 ± 0.14 a	0.18 ± 0.11 C - a
Ca (OH)_2_ + CHX	15.48 ± 9.1 a	14.63 ± 2.84 a	103.09 ± 21.0 A - b	0.24 ± 0.08 a	0.18 ± 0.07 a	0.33 ± 0.11 BC - a

Ca (OH)_2_: calcium hydroxide; SS: saline solution CHX: 2% chlorhexidine gel; Control group: pyrogen-free saline solution; S: Sample; SD: standard deviation. Different uppercase letters indicate inter-groups statistically significate differences (comparison in the same column), while different lowercase letters indicate intra-groups statistically significate differences (comparison in the same line).

## Discussion

The endodontic treatment classically is composed of two synergic concepts known as cleaning and shaping using mechanical instruments ^15^ and chemical substances ^16^ to disinfect the root canal system and detoxify the endotoxins ^14^. There is no doubt about the efficacy of the mechanical instrumentation using rotary or reciprocating files to reduce the microbial load and detoxify endotoxins including LPS and LTA ^17^. In the present study, low or even undetectable amounts of TNF-α, IL-1β, IL-6, G-CSF, nitric oxide, MIP-1α and IP-10 were observed immediately after the instrumentation in S1. Martinho et al. in their study confirm that the instrumentation of the root canal system detoxify endotoxins ^17^ and, thus, this will reduce the production of pro-inflammatory cytokines ^18^.

In this study, the use of sodium hypochlorite was avoided because of its elevated efficacy over diverse endodontic pathogens and their byproducts ^16^, and over the matrix metalloproteinases ^19^. The irrigant of choice was saline solution as it plays a neutral role during the instrumentation over pathogens and endotoxins. This neutral role is essential to obtain more accurate results about the effect of the medications over the evaluated pro-inflammatory cytokines.

Calcium hydroxide was effective in reducing pro-inflammatory cytokines production after 14 days. Similarly, in a recent study, Ca (OH)_2_ was effective in reducing the production of IL-1β, IL-6 and IL-10 after 7 days in an in vitro study ^20^. In another study, again calcium hydroxide-based intracanal medications were able to reduce the production of TNF-α and IL-1β in an in vitro study ^21^. 

In the present study, the best results were obtained with calcium hydroxide when compared to the other evaluated medications to neutralize the cytotoxic effects of LTA to induce the production of most of the evaluated cytokines (TNF-α, IL-1β, IL-6, G-CSF, MIP-1α) and nitric oxide by macrophages (RAW 264.7) with a statistically significant difference with the control group, except for the chemokine IP-10. Wang et al. ^22^ demonstrated that the pretreatment of LTA with calcium hydroxide inhibits its ability to induce the release of TNF-α, nitric oxide and MIP-1α in macrophages (RAW 264.7), because deacylated LTA cannot stimulate the receptor TLR2, and for this reason it is unable to stimulate the expression of pro-inflammatory cytokines. Ryu et al. ^23^ also found that the ability of LTA to induce nitric oxide and TNF-α was abolished when LTA was treated with 0.2 N sodium hydroxide, demonstrating that the alkaline treatment can inactivate LTA. As it happens on LPS, calcium hydroxide promotes detoxification of LTA, resulting in the release of free fatty acids and the loss of their immunostimulatory activity ^10^. Therefore, the available evidence seems to point that, when used as intracanal medication for 14 days, calcium hydroxide was effective in reducing the cytotoxic effects of LTA in macrophages.

Conversely, chlorhexidine was able to reduce the production of IL-6, G-CSF and nitric oxide after 14 days. In another study, CHX was as effective as Ca (OH)_2_ over TNF-α and IL-1β ^21^. Lee et al. ^24^ showed that pretreatment of LTA with 2% CHX for 6 hours or with 0.2% CHX for 24 hours caused a significant decrease in its ability to induce the production of TNF-α in macrophages (RAW 264.7), via receptor TLR-2. CHX can connect both LTA (by electrostatic interactions) and LPS (hydrophobic interactions) preventing cellular activation via TLR2 and TLR4, respectively ^25^. However, according to the results of this study, it was observed that chlorhexidine can neutralize some of the cytotoxic effects of LTA in macrophages, such as the ability of induction of the production of IL-6, G-CSF and nitric oxide, but not TNF-α, IL-1β, IP-10 and MIP-1α. Thus, it can be observed that the chlorhexidine, when used as an intracanal medication, was unable to promote effective neutralization of LTA at all the pro-inflammatory cytokines, with the need to expand the studies on the role of chlorhexidine on LTA and intracellular signaling pathways involved in the production of these cytokines and chemical mediators.

The combined intracanal medication of Ca (OH)_2_ and CHX was effective in neutralization of the cytotoxic effects of LTA resulted in a significant reduction of TNF-α, IL-6, G-CSF, IP -10 and nitric oxide production being similar to the Ca (OH)_2_ + SS group and different of the control group. Similarly, in a recent study, Ca (OH)_2_ + CHX was effective in reducing the production of IL-1β, IL-6 and IL-10 after 7 days in an in vitro study ^20^. Clinically, the combined effect of this intracanal medication had no significant complementary role in the reduction of endotoxins ^14^, however, in that study, sodium hypochlorite and other irrigants were used before intracanal medication.

An important point to be emphasized here is that despite the S3 were collected immediately after instrumentation have induced low production of cytokines, demonstrating effectiveness of the instrumentation protocol, it was found that in the control group, which received only saline during the period of 14 days of the medication, there was a significant increase in the production of all the evaluated cytokines (TNF-α, IL-1β, IL-6, G-CSF, MIP-1α and IP-10) and nitric oxide (p<0.5) in S3. Therefore, it was verified that LTA was also present inside the root canals system, requiring its neutralization with the use of effective intracanal medications. Along similar lines, these results agree with Baik et al. ^2008^, who reported that LTA has a high affinity to hydroxyapatite, which can accumulate and reside persistently inside the dentin tubules ^8^.

The combined use of Ca (OH)_2_ and CHX was suggested anteriorly in the literature ^6^ ^,^ ^14^ ^,^ ^19^. However, its combined effect on LTA was little explored ^14^. In this study, it was found that their combined effect results in favorable anti-inflammatory action as it reduced the production of different proinflammatory cytokines. 

The last point to be emphasized is the period of the intracanal medication remained in the canals. For Martinho et al. ^6^, who tested different 7- and 14-days intracanal medications on inflammatory cytokines in in vitro study, it was found that the 14-days intracanal medication is more effective than the 7-days one. In the present study, only one period was evaluated respecting the elevated efficacy of the 14-days protocol reported in the literature ^6^ ^,^ ^7^.

## Conclusions

The combined intracanal medication of calcium hydroxide and chlorhexidine is effective in reducing the cytokines TNF-α, IL-1β, IL-6, G-CSF and nitric oxide.
